# Why is Complexity Science valuable for reaching the goals of the UN 2030 Agenda?

**DOI:** 10.1007/s12210-020-00972-0

**Published:** 2021-01-27

**Authors:** Pier Luigi Gentili

**Affiliations:** Chemistry, Biology, and Biotechnology Department, University degli Studi di Perugia, Via Elce di sotto 8, 06123 Perugia, Italy

**Keywords:** Complex Systems, Networks, Out-of-equilibrium thermodynamics, Emergence, Computational Complexity, Natural Computing

## Abstract

The goals and targets included in the 2030 Agenda compiled by the United Nations want to stimulate action in areas of critical importance for humanity and the Earth. These goals and targets regard everyone on Earth from both the health and economic and social perspectives. Reaching these goals means to deal with Complex Systems. Therefore, Complexity Science is undoubtedly valuable. However, it needs to extend its scope and focus on some specific objectives. This article proposes a development of Complexity Science that will bring benefits for achieving the United Nations’ aims. It presents a list of the features shared by all the Complex Systems involved in the 2030 Agenda. It shows the reasons why there are certain limitations in the prediction of Complex Systems’ behaviors. It highlights that such limitations raise ethical issues whenever new technologies interfere with the dynamics of Complex Systems, such as human beings and the environment. Finally, new methodological approaches and promising research lines to face Complexity Challenges included in the 2030 Agenda are put forward.

## Introduction

The Heads of State and Government and High Representatives, meeting at the United Nations Headquarters in New York in September 2015, have made a historic decision on a comprehensive, far-reaching and people-centered set of global Sustainable Development Goals and targets (UN General and Assembly 2015). The Goals and targets, included in the 2030 Agenda, want to stimulate action in areas of critical importance for humanity and the Earth. These goals and targets regard the twenty-first century challenges that are global because they involve everyone on Earth under both the health and economic and social points of view (Harari 2018; Martin 2007; Lufkin 2017; Royal Geog. Society 2020).

The current pandemic (COVID-19) caused by a novel coronavirus (SARS-COV-2) is a concrete example of what is a global challenge (Frazer 2020). Similar examples are the epidemics of AIDS, tuberculosis, malaria, and neglected tropical diseases, hepatitis, waterborne and other communicable diseases. All these cases are included in the third goal of 2030 Agenda, which regards good human health and well-being.

Other examples of the twenty-first century challenges, included in the 2030 Agenda, concern about the Economy. The overarching goal of the United Nation is to originate a truly Sustainable Macro-Economy by transforming all the productive activities from linear to circular. This target is reachable if goods that are at the end of their service life are turned into resources for others, minimizing waste (Stahel 2016). Natural resources must not be exhausted through a responsible production and consumption, which is the content of the 12th goal of the 2030 Agenda. The access to affordable, reliable, sustainable, and modern energy should be ensured to every nation by promoting the exploitation of renewable energies, according to the 7th goal of the 2030 Agenda.

Manufacturing processes and all the other human activities should not perturb the fragile stability of natural ecosystems, as declared in the 2nd (regarding agricultural productivity), the 9th (regarding industrial production), the 11th (regarding the cities and human settlements), and the 14th (regarding fishing and the marine ecosystems) goals of the 2030 Agenda. The biodiversity of marine (14th goal) and terrestrial (the 15th goal) ecosystems should be carefully preserved. The climate change requires urgent interventions as stated in the 13th goal of the 2030 Agenda.

Economic and political choices should eradicate poverty from the Earth (according to the first goal) and avoid crises, such as the financial bubbles. The annihilation of poverty’s pockets in the world would promote social stability both globally and locally. The probability of wars, social unrest, and uncontrolled migrations would be reduced significantly.

Other relevant challenges regard justice in our societies. All the fundamental citizens’ rights should be defended (the 10th goal), whatever is their class, age, gender (included in the 5th goal), and health state. Examples of these rights are inclusive and equitable quality education (included in the 4th goal); availability of water and sanitation for all (declared as the 6th goal); full and productive employment and decent work for all adults, avoiding slavery and forms of child labor (included in the 8th goal); access to justice for all as mentioned in the 16th goal. The World Wide Web has revolutionized the diffusion of news and opinions, transforming the entire world into a global agora. Our democracies and free wills are at risk now more than ever due to the possible spreading of fake news. Aware of the fact that we are all strictly interconnected in this world, the accomplishment of the goals and targets of the 2030 Agenda requires the involvement of every nation and every citizen (as declared in the 17th goal).

In a nutshell, all the challenges that humanity is facing regard:

1. every human being and his psycho-physical well-being that depends primarily on his brain and immune system;
2. the human societies;
3. the world economy;
4. the ecosystems and the environment;
5. the climate of the Earth.

This is a list of Complex Systems. They are apparently so diverse and they are traditionally investigated by well-distinct disciplines. The Complexity Science has the ambitious aims of

- pinpointing the features shared by all those Complex Systems;
- delving more deeply their knowledge;
- spreading their interdisciplinary insight among all the members of our societies, and in particular the young generations, researchers, teachers, and the public and private managers.

The purpose of this paper is to advocate that Complexity Science can play a relevant role in accomplishing the 17 goals fixed by the members of the United Nation in the 2030 Agenda. This paper is structured as follows: Sect. 2 presents the features shared by the Complex Systems listed above; Sect. 3 explains the reasons why it is difficult to predict the behavior of those systems; Sect. 4 shows that our limitations in scientific predictability make many cutting-edge technologies highly disputable and raise ethical issues. Finally, Sect. 5 proposes strategies to investigate the Complex Systems more in depth for helping humanity to reach the goals of the 2030 Agenda. Section 6 reports conclusions and perspectives.

## The features of Complex Systems

What are Complex Systems? There is no commonly accepted definition of what a Complex System is, and thus, there are many different perspectives and opinions on the subject (Newman 2011; Mitchell 2009; Badii and Politi 1997; Johnson 2009; Gell-Mann 1995; Caldarelli 2020; Charbonneau 2017; Amaral and Ottino 2004; Corning 1998; Crutchfield and Machta 2011; Ladyman et al. 2013; Gentili 2018a; Ladyman and Wiesner 2020). We might claim that every discipline has its definition of Complexity. However, the Complexity Science has defined its areas of interest (Newman 2011). In physics and chemistry, examples of Complex Systems are superconductors, hydrodynamical fluids, chemical oscillators, and Turing patterns. In biology, every form of life is complex, even if it is unicellular. Multi-cellular organizations such as the nervous and the immune systems are complex, as well. Of course, communities of living beings are also complex. These communities can be composed of members of the same species, and they can give rise to spontaneous phenomena of self-organization, such as bacterial colonies, flocking, schooling, and migrations. The co-existence of different species in the same environment generates complex ecosystems. The most complex living beings are undoubtedly humans. Humans are the most populous large mammal on Earth, reaching 7.8 billion individuals. Human activities are so pervasive that they seem to perturb the Earth’s complex climate, and the current geological epoch has been named Anthropocene. Humans give rise to complex social structures, networks, and urban areas. The interactions between manufacturers, traders, and consumers originate the complex world economy.

All these examples of Complex Systems appear very different, and distinct scientific disciplines traditionally investigate them. One purpose of the Complexity Science is to point out their common features. In this work, we propose four attributes that, we believe, are shared by all the mentioned Complex Systems, in particular by those involved in the goals of the 2030 Agenda and listed in the Introduction of this article.

### Networks

All Complex Systems can be represented and described as networks. The representation of the Complex Systems as networks is also suggested by the etymology of the word “Complex”. The adjective “Complex” derives from the Latin verb “cum-plectere” which means “to intertwine together”. It is different from the etymology of the word “complicated”, even though “complex” and “complicated” are considered synonyms in the colloquial language. The adjective “complicated” derives from the Latin verb “cum-plicare”, whose meaning is “to fold together”. What is “complicated” is “folded” and can be “unfolded”. On the other hand, what is “complex” is “interwoven” and it cannot be “unfolded”: it must be “untangled” (Gentili 2018a, b).

Networks are constituted by nodes and edges or links (Newman 2010). The nodes are the elements of the network, whereas the edges are the relationships among the nodes. The network representation can be applied to all Complex Systems. Superconductors, hydrodynamical fluids, chemical oscillators, and Turing patterns appear as continuous at the macroscopic level, but they can be described as networks at two different spatial scales. At the atomic scale (when the spatial dimensions are in between Å and tens of Å), every constitutive molecule plays as a node of a network whose links are the intermolecular forces. At the microscopic level (when the spatial scale is in between tens and hundreds of nm), these continuous systems are representable as a grid of mesoscopic dynamical systems interplaying reciprocally. Even all those Complex Systems that are the focuses of the 2030 Agenda goals can be described as networks (see Table 1). In a society, there are a huge number of possible networks, wherein the nodes are the individuals or groups of them, and the links are their mutual relationships, such as friendship, communication, family (Freeman 2004; Wasserman and Faust 1994). In the world economy, there is a large family of different networks, which stems from the fact that there are different building blocks for defining the nodes in an economic network (Smith and White 1992; Hughes and Nagurney 1992). For instance, if the nodes are the countries, the economic edges can be their trading relations (Emmert-Streib et al. 2018). In the nervous system, the nodes might be the single neurons and the links are the synaptic connections (Telesford et al. 2011). In the immune system, the basic nodes are the immune cells and the links are the signaling molecules, such as the cell-surface receptors and secreted molecules (Rieckmann et al. 2017; Shi et al. 2020). In biological ecosystems, the species are the nodes and their trophic and symbiotic interactions are the edges (Dunne et al. 2002; Krause et al. 2003). The climate can be represented as a network if the nodes are identified as the sites in a spatial grid of the underlying global climate dataset and the edges are related to the degree of statistical similarity (that may be related to dependence) between the corresponding pairs of time series taken from climate records (Tsonis and Roebber 2004; Donges et al. 2009).

**Table 1:** Examples of Complex Systems described as networks with nodes and links

Complex Systems	Nodes	Links
Human societies	Individuals	Mutual relationships
World economy	Countries	Trading relations
Nervous system	Neurons	Synaptic connections
Immune system	Immune cells	Signaling molecules
Biological ecosystems	Species	Symbiotic and trophic relations
World climate	Sites of a spatial grid	Correlations of the time series

Beyond the number of nodes and edges, two other properties are valuable to describe a network quantitatively. One is the distance between nodes, which is measured by counting the number of links we need to pass through to go from one node to another. The most interesting path is that having the shortest distance (${\text{sp}}$), i.e., the path with the smallest number of links between the selected nodes. The average of the shortest paths between all pairs of nodes, named as “mean path length” ($\overline{{{\text{sp}}}}$), gives an idea of the overall navigability of a network. The other property that is useful to characterize a network is its clustering. The clustering coefficient $C$ of a node represents the fraction of pairs of neighbors that are connected to one another. The average clustering coefficient, $\overline{C}$, of a network quantifies the tendency of the nodes of that network to form clusters.

After an analysis of the properties of several real networks, six principal architectures have been proposed so far (see Table 2). One architecture is that of “regular networks” (or “lattices”). As the same name suggests, in a regular network, every node has the same degree. The degree distribution $P\left( d \right)$ is a delta-Kronecker function. The mean path length for a D-dimensional lattice with N nodes is long and equal to $\overline{{{\text{sp}}}} \sim N^{1/D}$ (Amaral and Ottino 2004). The clustering is high. The randomization of a fraction of the links in the “lattice” determines a significant reduction of $\overline{{{\text{sp}}}}$. Therefore, the “regular network” transforms in a “small-world network” (Watts and Strogatz 1998). The substitution of short-range edges with long-range ones creates shortcuts between nodes that would otherwise be much farther apart. In a “small-world network”, $\overline{C}$ maintains high and $P\left( d \right)$ is still a discrete function. The complete randomization of the links of a “lattice” generates a “random network”. In a “random network”, $P\left( d \right)$ is a Poisson function peaked at the average value of the degree $\overline{d}$, and decaying exponentially for $d \gg \overline{d}$ (Erdös and Rényi 1960). Furthermore, $\overline{{{\text{sp}}}}$, which is proportional to the logarithm of the number of nodes ($\overline{{{\text{sp}}}} \sim {\text{log}}(N)$), is shorter than that of a “small-world network”; the clustering coefficient is low and independent of the nodes’ degrees (Barabási and Oltvai 2004). $\overline{C}$ decreases as $N^{ - 1}$, i.e., the number of nodes in the network. Another possible architecture model is the “scale-free network”. Its degree distribution approximates a power-law of the type $P(d)\sim d^{ - \gamma }$, with $\gamma$ being a positive constant, usually included between 2 and 3 (Barabási and Oltvai 2004). This degree distribution implies that most nodes have low degree, and just a few have high degrees. The nodes that are highly interconnected are called hubs. The power-law distribution appears as a straight line on a log–log plot (see Table 2). The “scale-free networks” have a $\overline{{{\text{sp}}}} \sim {\text{log(}}\log (N){)}$ that is significantly shorter than those characterizing “random and small-word networks”. Finally, $C\left( d \right)$ is high and independent of $d$. If a network shows distinct groups of nodes, it is a “modular network” (see the graphical example of Table 2). Nodes within each module are highly interconnected, whereas nodes bridging distinct modules have low degree. The degree distribution of a “modular network” is discrete: most of the nodes have high degrees and just a few sparse interconnectivity. The average clustering coefficient is high. When clusters of nodes are combined in an iterative manner, a “hierarchical network” is generated (see the example of Table 2). In a “hierarchical network”, both $P\left( d \right)$ and $C\left( d \right)$ are power-law functions (Ravasz and Barabási 2003): nodes that appear in the center of the network have the largest degree and the smallest clustering coefficient, while the nodes at the periphery have low degrees and large clustering coefficients.

**Table 2:**
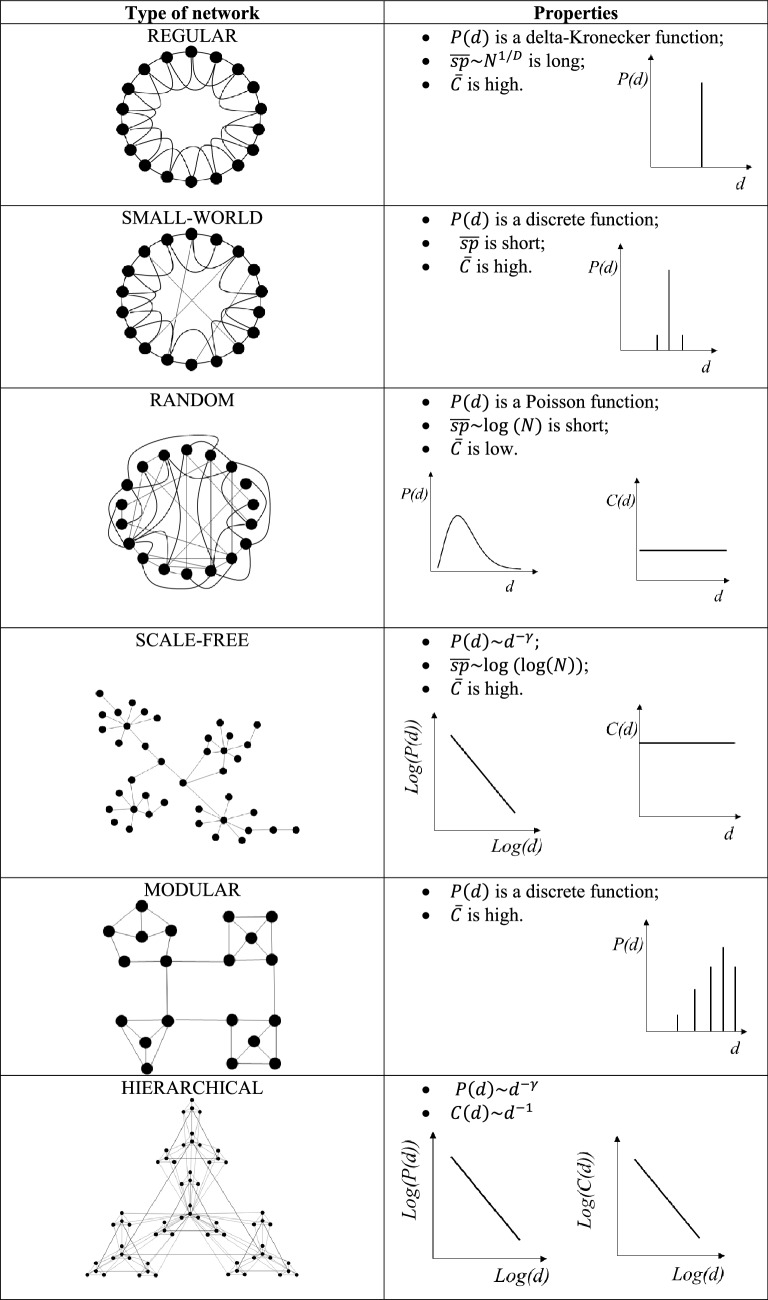
Types of networks architectures and their properties

Real complex networks are difficult to be studied (Strogatz 2001; Barabási 2014; Caldarelli and Catanzaro 2012; Caldarelli and Vespignani 2007). They might be intricate and challenging to be untangled. The connections might have different strengths and effects; nodes are often diverse. Moreover, real networks are dynamic: both the links and the nodes’ internal states can change non-linearly in time. The changes in the nodes’ features influence the types and strengths of the links and vice versa. Despite all these difficulties, the investigation of the properties of the Complex Systems, in fields ranging from cell biology to business and climate, has so far revealed that many of them show two attributes of the “small-world networks”: a short mean path length and a high clustering coefficient (Albert and Barabási 2002). “Small-world networks” guarantee an enhanced signal-propagation speed, computational power, synchronizability, and fast spread of epidemics and fads (Watts and Strogatz 1998). The degree distributions of the complex networks are often power-law $P\left( d \right)\sim d^{ - \gamma }$. This evidence demonstrates that many Complex Systems are “scale-free networks”. Their diffusion is due to the fact that real networks tend to grow continuously, adding always new nodes. The new nodes connect most likely to the nodes that already have many links, according to a process known as preferential attachment (Barabási and Albert 1999)*.* The overall topology of the “scale-free networks” is robust against random failures that involve most likely nodes with low connectivity, whereas it is vulnerable to attacks that regard hubs. The removal of a key hub splinters the original network into smaller clusters of nodes (Albert et al. 2000). Finally, it has been discovered that real networks that are scale-free and devoid of topological constraints, such as limitations in the links’ length, have hierarchical topology. Distinct modules are connected hierarchically, transforming their clustering coefficient in a power-law function of the degree: $C\left( d \right)\sim d^{ - 1}$ (Ravasz and Barabási 2003).

### Out-of-equilibrium

Complex Systems are out-of-equilibrium in the thermodynamic sense. Most of them are open systems because they exchange both matter and energy with the surrounding environment. There is probably one example of a Complex System that is isolated, and it is our universe, which is postulated to exchange neither energy nor matter. The universe is maintained far-from-equilibrium by its large internal gradients.

If a Complex System is constituted only by inanimate matter, its behavior is driven by force fields. On the other hand, if a Complex System also involves living beings, its behavior depends on not only the force fields but also the information. As far as we know, life, as we conceive it, is present only on Earth, and despite the mesmerizing variety of life forms, it is possible to pinpoint some features shared by all living beings. They are summarized below and embedded into three sets regarding (1) the variable information, (2) life cycle, and (3) adaptation–acclimation–evolution, respectively.

(1) Information variable: The first peculiarity of living beings is that of exploiting matter and energy to encode, collect, store, process, and send information (Roederer 2005; Walker et al. 2017). All living beings are “Information Gathering and Utilizing Systems (IGUSs)” due to their ability to process the messages about the environment, to make decisions on their actions (Gell-Mann 1994; Zurek 1990; Wheeler 1990). Information is used by living beings to reach their purposes. The basic aims common to every living being are those of surviving and reproducing. This quality is called teleonomy (Monod 1971).

(2) Life cycle: Every living being is an open system, characterized by a boundary that delimits it from the rest of the environment. It has a birth, a life during which it grows and ages. It is able to self-maintain and self-reproduce, according to the autopoiesis theory (Maturana and Valera 1980). It has the power of protecting itself from some intruders and harmful elements. However, finally, it dies when all fundamental internal activities cease.

(3) Adaptation–acclimation–evolution: At the cellular level, the information required to live is preserved into the DNA. However, the DNA is not a blueprint of a living being because the sequence of genes does not have a one-to-one correspondence with all its constitutive elements. Instead, a genome is an algorithm (Davies 2012) for building a living being, and a large amount of information for its development comes from the environment. During its lifetime, every living being is capable of adapting by adjusting its metabolic processes, acclimating by expressing new genes, evolving by changing its genome under an ever-changing environment (Rojdestvenski et al. 2003).

According to the theory of out-of-equilibrium thermodynamics, living beings are dissipative structures that maintain order within them by dissipating energy and releasing entropy in the surrounding environment (Prigogine and Lefever 1973). The evolutionary criteria for the out-of-equilibrium systems are under scrutiny. What maintains a system out-of-equilibrium is the presence of one or more forces ($F_{i}$), which generate fluxes ($J_{i}$). The sum of the products of the conjugated forces and flows defines the entropy production ($P^{*}$) of any out-of-equilibrium system:

1 $$ P^{*} = \frac{{{\text{d}}_{i} S}}{{{\text{d}}t}} = \mathop \sum \limits_{i} J_{i} F_{i} . $$

For instance, when the force is a thermal gradient $\left( {F_{i} = \vec{\nabla }\left( \frac{1}{T} \right)} \right)$, the flow is the heat conduction $\left( {J_{i} = \frac{{{\text{d}}Q}}{{{\text{d}}t}}} \right)$ and the entropy production is

2 $$ P^{*} = \frac{{{\text{d}}_{i} S}}{{{\text{d}}t}} = \left( {\frac{{{\text{d}}Q}}{{{\text{d}}t}}} \right)\vec{\nabla }\left( \frac{1}{T} \right). $$

When the forces are constant, the system evolves up to reach a stationary state or a self-organized critical and dissipative state, where $P^{*}$ extremizes (Kondepudi and Prigogine 2015). For systems that work in the linear regime, i.e., when the relationships between flows and forces are linear ($J_{i} = \sum\nolimits_{k} {L_{i,k} } F_{k}$ being $L_{i,k}$ the proportionality constant between the flow $J_{i}$ and the force $F_{k}$), $P^{*}$ reaches a minimum at the stationary state:

3 $$ {\text{d}}P^{*} \le 0. $$

On the other hand, for systems that are very far from equilibrium, i.e., in the nonlinear regime (when the flows are nonlinear functions of the forces), it seems that a general evolutive criterion is instead the maximization of $P^{*}$ (Martyushev and Seleznev 2006):

4 $$ {\text{d}}P^{*} \ge 0. $$

The Maximum Entropy Production Principle is relatively new, and its range of applicability is not fully understood (Martyushev 2013). Probably, it might help to bridge the gap between the evolution of inanimate and animate systems (Goldenfeld and Woese 2011). It is worthwhile pursuing the thermodynamic description of Complex Systems for different reasons. First, thermodynamics is indispensable when the phenomena under investigation determine matter and energy transformations. Complex Systems and living beings exist in a world of energy and material fluxes. Life is a far-from-equilibrium system that maintains its local level of organization at the expense of the larger global entropy budget (Schrödinger 1944). Second, thermodynamics is the only scientific theory that contemplates irreversibility through the variable entropy (Kondepudi and Prigogine 2015). Third, it is interdisciplinary, being applied in physics, chemistry, biology, ecology, economy, and sociology. Finally, thermodynamics is expected to extend its scope by including the variable information under the quantitative and qualitative point of view. Such supposed development will allow a deeper understanding of living matter (Gentili 2018a, b; Dittrich 2015).

### Emergent properties

**Fig. 1:**
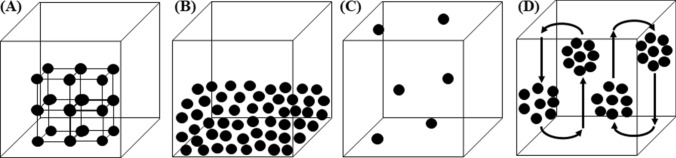
Schematic representations of a solid (**a**), liquid (**b**), gas (**c**) phase, and a fluid with convective motion (**d**). The molecules (black dots) are the nodes of the networks and their intermolecular forces are the corresponding edges

Any Complex System is a network that exhibits one or more emergent properties. Emergence comes from the Latin “ex mergere”, which means to turn up, to appear as a result. Emergent properties come to light from the ensemble of the nodes and their relationships. The integration of the nodes’ features and their links gives rise to properties that belong to the whole network. The whole network is “more than the sum of its parts”, as properly alleged by Aristotle in his Metaphysics (Annas 1976). “The whole is not only greater than but very different from the sum of the parts”, as declared by Anderson, who participated in the founding workshops of the Santa Fe Institute, in his seminal paper (Anderson 1972) written at the dawn of the Complexity Science’s development. In the history of philosophy and science, different taxonomies of emergent properties have been proposed (Bar-Yam 2004; Clayton and Davies 2006; Corning 2002). A more recent classification is based on the idea that Complex Systems can be represented as networks, and every type of network originates its peculiar emergent properties (Gentili 2018a, b). For instance, the inanimate matter that is at the thermodynamic equilibrium and at a specific temperature and pressure, can give rise to crystalline solid phases when its nodes, i.e., its molecules, are tightly bound and form a regular network (see Fig. 1a). If the molecular network is less regular because its nodes are linked through looser forces and do not have fixed spatial positions, it gives rise to a liquid phase (Fig. 1b). In the case of an even less tight network, due to weak intermolecular forces, the gas phase emerges (Fig. 1c). When the inanimate matter is far from the thermodynamic equilibrium, phenomena of temporal and spatial self-organization, but also chaotic dynamics can emerge. Examples are the oscillatory chemical reactions, chemical waves, Turing patterns, periodic precipitations, and convection of fluids that can be either ordered or turbulent (Gentili 2018a, b). In terms of networks’ topology, we might state that the symmetry breakings that occur when these phenomena of self-organization and chaos pop up are induced by transitions of the molecular networks from regular to modular ones. For instance, convection starts when large vertical thermal gradients promote the organization of molecules in micrometric clusters that move either upward or downward, according to Rayleigh’s model (see Fig. 1d). The molecular clusters or modules at higher temperatures and smaller densities move upward, whereas the molecular clusters or modules at lower temperatures and larger densities move downward. The micrometric molecular modules that interplay when they are physically in contact, spontaneously self-organize in a collective motion that gives rise to a macroscopic pattern of roll-shaped cells, having a long tubular geometry. Adjacent cylindrical rolls rotate with the same velocities, but in opposite directions: one clockwise and the other anti-clockwise. The fluid flow is laminar. When the thermal gradient becomes very large, a turbulent flow emerges wherein the microscopic modules have distinct velocities and are more independent: they do not move collectively in an ordered manner.

Life is one of the most, if not the most, astonishing instances of an emergent property. The molecular constituents of every living being, are phospholipids, water, proteins, RNA, DNA, and salts. Taken separately, they never show the phenomenon of life. Life emerges only if all the necessary molecular constituents are organized in that peculiar hierarchical network structure, which is a cell (Barabási and Oltvai 2004). In any living being describable as hierarchical network (Alcocer-Cuarón et al. 2014), both upward and downward causations are possible. Upward causation is when the features of lower levels determine the emergent properties of the higher levels. A trendy and dramatic example is offered by the coronavirus SARS-CoV-2 that, after jumping into a human being, has triggered the pandemic COVID-19. A microscopic agent, such as the SARS-CoV-2 virus, replicates and invades an entire organism after infecting a cell. Then, the infection can spread among individuals, societies, nations, and affects our lives from both the medical and social, and economic points of view. Downward causation is when the properties and events of higher levels influence those of the lower levels. An example is the act of eating food that ends in influencing intracellular events. A key element for generating top–down causation in a living being is the fractal-like structure of chromatin. The chromatin’s behavior is influenced by both the genes it has and the forces exerted on it from the rest of the cell and the cell’s environment (Davies 2012). The possibility of having both downward and upward causation gives living beings the power to adapt, acclimate, evolve, and influence their environment. Living beings and their societies are Complex Adaptive Systems (Miller and Page 2007).

Groups of living creatures can organize in different network architectures and show peculiar emergent properties. For instance, there are certain types of fishes, such as the bigeye trevallies (*Caranx sexfasciatus*) that organize in school, and certain types of birds, such as the snow geese (*Chen caerulescens atlantica*), that assemble in flocks. Any school of fish or any flock of birds can be described as a network whose structure is comparable to that of a hydrogel. Schools of fishes and flocks of birds exhibit the emergent property of swarm intelligence, which is a collective and decentralized intelligent behavior (Bonabeau et al. 1999). The swarm intelligence derives from the interactions among the individuals or agents that follow a code of three simple rules based on local information. The first is the alignment rule: an individual assumes a velocity close to the mean value of its nearest neighbors. The second is the cohere rule: after alignment, an individual takes a small step in the direction that the center of mass of the group chooses. The third is the separate rule: an agent should always avoid any collision.

Social insects, such as the bees, organize in colonies, whose structures have some features of the scale-free networks (Fewell 2003). In a hive, the most apparent hub is the queen: she does not centrally control all the colony functions, but, in honeybees, she secrets a pheromone that represses reproduction in workers and maintains the colony’s cohesion. There are other hubs within the worker task groups: they are the scouts or dancers. Their essential role is to communicate the food location and availability, which assures the cohesion of the bees that go out to forage. As in any scale-free network, the removal of hubs disrupts the network severely, whereas the loss of any other type of nodes would have a much smaller effect.

Finally, the symbiotic and trophic relationships that can be encountered in any ecosystem give rise to modular networks. Each module represents a biological species. The cooperative or competitive actions between distinct species originate emergent properties. One relevant example is the biological macroevolution. The ecosystem as a whole evolves to a “self-organized nonequilibrium state”, where periods of stasis alternate with avalanches of causally connected evolutionary changes (Sneppen et al. 1995). The number of avalanches of a given size *s* obeys to a power-law of the type $N\left( s \right) \approx s^{ - 1.1}$ based on fossil record. Avalanches of all sizes occur, including large catastrophic ones, with no needs of external causes, such as climate change or the fall of meteorites. The possibility of having extinctions of all sizes is an emergent property of ecosystem dynamics.

### Towards a formal definition of Natural Complexity

After describing the principal features shared by all the Complex Systems, it is spontaneous to propose a formal definition of Natural Complexity, which is expected to be cross-disciplinary because it might be applied in distinct disciplines. Natural Complexity (${\text{NaC}}$) is a multivariable function that depends on:

1. The Multiplicity (${\text{Mu}}$) of the network, which is the number $N$ of nodes. The nodes are described by the temporal functions of the type: $x_{1} \left( t \right), x_{2} \left( t \right), \ldots , x_{N} \left( t \right).$
2. The Interconnection (${\text{Ic}}$) of the network, which is the number $L$ of the links among the nodes. All the potential edges can be represented by the $N \times N$ adjacency matrix $A$ that describes the system’s wiring and the interaction strength between the nodes:

5 $$ A = \left( {\begin{array}{*{20}c} {a_{11} } & \cdots & {a_{1N} } \\ \vdots & \ddots & \vdots \\ {a_{N1} } & \cdots & {a_{NN} } \\ \end{array} } \right) $$

3. The Diversity of the nodes (${\text{Di}}_{{\text{N}}}$) when the functions, $x_{1} \left( t \right), x_{2} \left( t \right), \ldots , x_{N} \left( t \right)$, are not the same.
4. The Diversity of links (${\text{Di}}_{{\text{L}}}$) when the elements of the matrix $A$ are different.
5. The Variability of the nodes (${\text{Va}}_{{\text{N}}}$), which depends on how much the functions $x_{1} \left( t \right), x_{2} \left( t \right), \ldots , x_{N} \left( t \right)$ evolve in time.
6. The Variability of the links (${\text{Va}}_{{\text{L}}}$), which depends on how much the coefficients of the adjacency matrix $A$ (i.e., $a_{11} \left( t \right)$, $a_{12} \left( t \right), \ldots , a_{NN} \left( t \right)$) change over time.
7. The Integration (${\text{Ig}}$) of the nodes’ and links’ features, which gives rise to those properties that are called emergent because they belong to the entire network. As it has been sustained in the previous paragraph, the emergent properties depend on the network’s architecture. The network’s topology can be inferred from parameters such as the degree distribution ($P\left( d \right)$), the clustering coefficient ($C$) of the nodes, and the mean path length ($\overline{{{\text{sp}}}}$) of the network. Furthermore, the emergent properties depend on the environment surrounding the system as proved by the Darwinian evolution of life (Adami et al. 2000).

In synthesis, Natural Complexity (${\text{NaC}}$) results to be a seven-variable function of the type:

6 $$ {\text{NaC}} = f\left( {{\text{Mu}}, {\text{Ic}},{\text{Di}}_{{\text{N}}} , {\text{Di}}_{{\text{L}}} , {\text{Va}}_{{\text{N}}} ,{\text{Va}}_{{\text{L}}} , {\text{Ig}}} \right). $$

The evaluation of ${\text{NaC}}$ should not be limited to just a few of the seven variables indicated in Eq. (6), but it should encompass all of them. In the literature, there are many definitions of Natural Complexity and each one refers to one or just a few of the variables appearing in Eq. (6). It is not satisfactory to focus on just some structural features (e.g., the degree distribution $P(d)$) and use the Shannon Entropy or the Kolmogorov Complexity to sort out different Complex Systems (Morzy et al. 2017). It is not adequate to limit our attention just on the emergent properties or functions of a system (i.e., its ${\text{Ig}}$), and use algorithms, such as the Functional Information, for measuring the degree of Complexity (Szostak 2003; Hazen et al. 2007)*.* In economy, a metrics of complexity based on the export baskets of the different countries (i.e., their “emergent goods and services”) has been proposed (Sciarra et al. 2020). The drawback of an incomplete Natural Complexity description is encountered even in sociology and ecology (McShea 1996). The complexity of both human and animal societies has been most often determined by measuring the number of relationships (${\text{Ic}}$) and/or the diversity of social relationships (${\text{Di}}_{{\text{L}}}$) (Kappeler 2019).

Accurate structural, functional, and dynamical analyses of any Complex System are often challenging tasks (Farine and Whitehead 2015). However, when the experimental access to a Complex System is limited, luckily, it has been demonstrated that a well-selected subset of variables can contain sufficient information about the rest. Such variables have been called sensors (Liu et al. 2013). Furthermore, if the final goal is the control of the Complex System behavior, it could be enough to identify the set of driver nodes that can guide the system’s entire dynamics (Liu et al. 2011).

## Predictability

All the Complex Systems, targeted by the United Nations and mentioned in the Introduction, share another common feature beyond those presented in the previous section. They cannot be described exhaustively. In other words, science finds insurmountable obstacles in predicting their behavior, especially in the long term. This feature outlines an operational definition of Complex Systems. There are three principal reasons why this definition holds. These reasons are presented in the next three subparagraphs.

### Computational Complexity

Most of the computational problems regarding Complex Systems are solvable but intractable. Examples are planning, scheduling, machine-learning, financial forecasting, design of computers’ hardware, solving the Schrödinger equation for determining molecular energies, the Traveling Salesman Problem (TSP) (Monasson et al. 1999), system identification (Cubitt et al. 2012), protein folding (Berger and Leighton 1998), etc. According to the theory of Computational Complexity (*CoC*) (Goldreich 2008), all the computational problems can be grouped into two sets: the set of Polynomial (*P*) problems and the set of Exponential problems. A problem is polynomial when the number of computational steps (${\text{n}}^\circ {\text{comp}}. {\text{steps}}$), required to find the exact solution of the problem, is a polynomial function of the dimension *N* of the problem:

7 $$ {\text{n}}^\circ {\text{comp}}{\text{. steps}} \propto N^{k} $$

with $$k = 1, 2, \ldots$$.

All the P problems are problems of recognition; they are solvable and tractable. It is possible to determine the exact solution in a reasonable lapse of time, whatever is the dimension (*N*) of the problem.

**Fig. 2:**
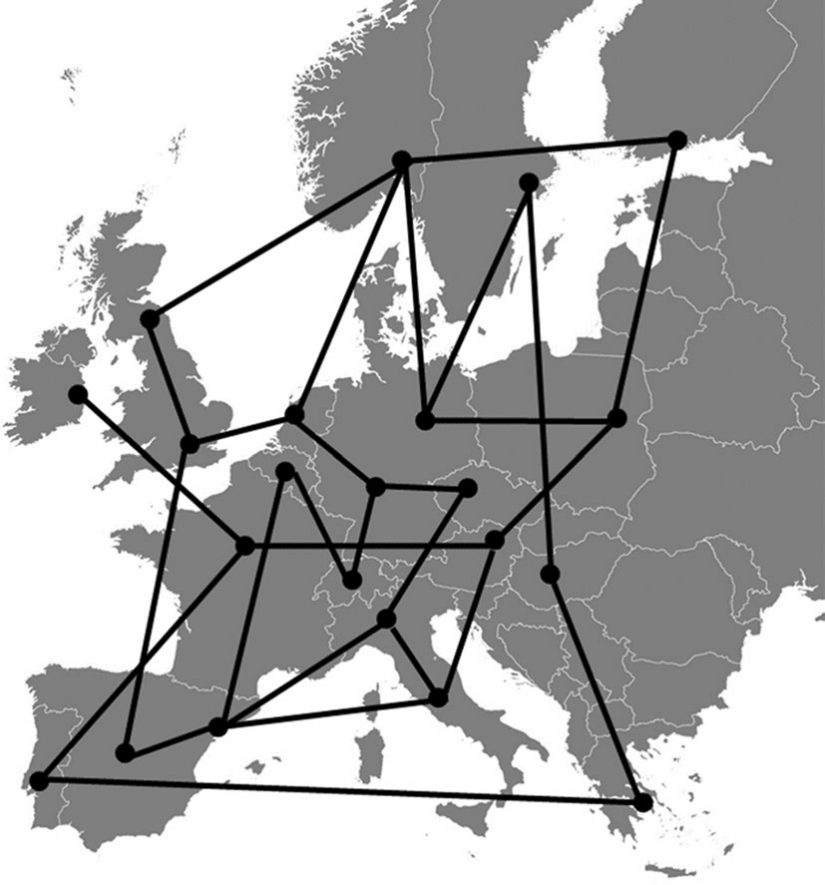
The solution of the TSP requires that, given a map with a certain number of cities, flights, and costs, the salesman finds the least-cost closed tour crossing each city just once

A problem is exponential when the number of computational steps is an exponential function of *N*. For instance, in the case of the Schrödinger equation, ${\text{n}}^\circ {\text{comp}}{\text{. steps}} \propto 2^{N}$, wherein *N* is the number of particles. Unfortunately, when we face exponential problems having large dimensions (i.e., large *N* values), it is impossible to find the exact solution in any reasonable lapse of time, whatever is the rate of our computing machines. In the case of the TSP, the ${\text{n}}^\circ {\text{comp}}{\text{. steps }} \propto N! \approx N^{N}$. The TSP requires that, given a graph with nodes (i.e., a map with cities), edges, and costs associated with the edges (i.e., connections and their costs), the least-cost closed tour containing each of the nodes just once is determined (see Fig. 2).

For a map with five sites, the maximum number of computational steps is $5! = 120$; if $N = 15$, ${\text{n}}^\circ {\text{comp}}{\text{. steps}} \approx 1.3 \times 10^{12}$; if $N = 100$, ${\text{n}}^\circ {\text{comp}}{\text{. steps}} \approx 9 \times 10^{157}$ (see Table 3). According to the TOP500 project (https://www.top500.org/), which ranks the 500 most powerful non-distributed computer systems in the world, twice a year, the fastest supercomputer in the world in June 2020 is the Japanese Fugaku. It reaches the astonishing computational rate of 415.5 PFlops/s (1 PFlops/s corresponds to $1 \times 10^{15}$ floating-point operations per second). With the availability of Fugaku, the time required to solve the TSP is really short when $N = 5$ or $N = 15$ (see Table 3). It becomes extremely long when $N = 100$: it is $2 \times 10^{ + 140} s$, which corresponds to $\approx 6.4 \times 10^{132} \;{\text{years}}$. This amount of time is unreasonable. Suffice to think that it is enormously longer than the age of the universe, which has been estimated to be $14 \times 10^{9} \;{\text{years}}$. It is evident that it is necessary to abandon the idea of finding the exact solutions of large exponential problems if the only possible algorithm is that of brute force.^1^

**Table 3:** The ${\text{n}}^\circ {\text{ comp}}{\text{. steps}}$ and ${\text{time}}$ required to solve the TSP assuming of having the Japanese supercomputer Fugaku to make the computations

${\text{n}}^\circ {\text{sites}}$	${\text{n}}^\circ {\text{comp}}{\text{. steps}}$	Time
5	120	$\approx 3 \times 10^{ - 16} s$
15	$15! \approx 1.3 \times 10^{12}$	$\approx 3 \times 10^{ - 6} s$
100	$100! \approx 9 \times 10^{157}$	$\approx 2 \times 10^{ + 140} s$

The exponential problems with large *N* are intractable. They need to be transformed in Non-deterministic Polynomial problems or NP-problems. In other words, plausible solutions are generated non-deterministically through heuristic algorithms, and they are evaluated after fixing an arbitrary criterion of acceptability. The original exponential problems are handled as if they were polynomial because they are reduced to recognize if the solutions, generated non-deterministically, verify the imposed conditions or not. Mathematicians, computer scientists, and polymath scientists are contriving brand-new heuristic algorithms to achieve acceptable solutions to the NP-problems in always shorter times. Meanwhile, others try to figure out if there exist algorithms that can reduce the NP to P problems, or this reduction is impossible. If the NP were reduced to P problems, humanity would become able to understand nature as never before. As Kurt Gödel declared in a letter to John Von Neumann, in 1956, the discovery that the NPs are reducible to P problems would have “consequences of the greatest magnitude” (Sipser 1992). Human life would not be the same. Everything would be much more efficient (Fortnow 2009). The transportation schedules would be optimized, allowing people and goods to move quicker and cheaper. Manufacturers and business people would improve their production processes and increase profits. It would become easier to find out a vaccine for pandemics, such as COVID-19, and new effective treatments for incurable diseases, make weather forecasts, predict catastrophic events, and the trends in stock markets. Humanity would have new valuable tools and methods to tackle the twenty-first century challenges. This is the reason why the Clay Mathematics Institute in Cambridge, Massachusetts, has named “*P* versus NP” as one of its “Millennium” problems. It offers one million dollars to anyone who provides verified proof that either ${\text{NP}} = P$ or ${\text{NP}} \ne P$.

### Variable patterns

Complex Systems exhibit variable patterns. Variable patterns are objects (both inanimate and animate) and events, whose recognition is made difficult by their multiple features, variability, and extreme sensitivity on the context. Examples of variable patterns are human faces and voices, fingerprints, handwritten cursive words and numbers, patterns and symptoms in medical diagnosis, patterns in apparently uncorrelated experimental data, aperiodic time series, political and social events. We need to formulate algorithms for recognizing every type of pattern, whatever is their context. The traditional steps followed to recognize variable patterns are: (1) data acquisition by instruments; (2) selection of the representative features of the pattern; (3) application of an algorithm for the classification step. So far, different statistical (Jain et al. 2000) and structural algorithms, along with artificial neural networks’ algorithms, have been proposed for recognizing variable patterns (Bishop 2006). All of them suffer in universality and effectiveness.

The difficulty in recognizing variable patterns and describing Complex Systems (Lloyd 2001) highlights a third type of complexity that we might name as Descriptive Complexity. The Descriptive Complexity is related to the total information needed to describe any Complex System and its variable patterns. Such information consists of two contributions, as outlined by the Nobel prize Gell-Mann and Lloyd (1996). The first contribution is the information needed to describe regular and rule-governed features, named as Effective Complexity. The second one is the information required to describe irregular and apparently random features, and it is connected to probability. It is useful to encode the description of the variable pattern into a bit string. For such string, we can make use of the concept of Algorithmic Information Content (AIC),^2^ which is a kind of minimum description length. The AIC of the variable pattern is the length of the shortest program that will allow a given universal computer to print out the string and then halt. The AIC of the string is split into two terms: one for regularities and the other for features considered as incidental or random. The sum of the two contributions can be defined as Descriptive Complexity (DEC). The minimum description length of the regularities of the pattern represents Effective Complexity. It is worthwhile noticing that the Effective Complexity is context-dependent and even subjective. It depends on the coarse-graining, i.e., the level of detail, at which the entity is described, the language used to describe it, the previous knowledge and understanding, and the distinction made between regular and incidental attributes (Gell-Mann and Lloyd 2003).

### The intrinsic limitations in the predictive power of science

Even if, one day, someone demonstrated that all the exponential problems are reducible to polynomial problems, i.e., ${\text{NP}} = P$, and universally valid and effective algorithms were formulated to recognize variable patterns, certain limitations in predicting the behavior of Complex Systems would remain.

As far as the microscopic world is concerned, the Uncertainty Principle holds. It was formulated, at first, by Heisenberg, and then, recently, extended and experimentally confirmed (Erhart et al. 2012). According to the Uncertainty Principle, it is impossible to determine accurately and simultaneously relevant variables of any microscopic particle, such as its position and momentum. This inaccuracy places limits to the deterministic dream of predicting the dynamic of the entire universe starting from the description of its ultimate constitutive particles.

We might think of limiting the description of Complex Systems at the macroscopic level, neglecting their microscopic constituents. However, Complex Systems can exhibit chaotic dynamics. Chaotic dynamics are aperiodic and extremely sensitive to the initial conditions (Feldman 2012). The experimental determination of the initial conditions is always affected by unavoidable uncertainties and experimental errors. Science is said to be exact not because it is based on infinitely exact data, but because its rigorous methodology allows estimating the extent of uncertainty associated with any quantitative determination. The inescapable uncertainty in defining any system’s initial conditions makes the chaotic dynamics unpredictable in the long term by definition.

## Bioethical Complexity

The intrinsic limitations in predicting the behavior of Complex Systems make many ethical issues that arise from the unstoppable technological development, highly disputable. There is a mutual positive feedback action between science and technology (Gentili 2018a, b). New scientific knowledge, sooner or later, promotes brand new technologies, and, vice versa, new technical achievements allow us to deepen our exploration of natural phenomena and hence gather finer data. The relentless technological innovations push humanity on the edge of always new ethical arguments and debates. All these ethical issues sprout from a universal fundamental question: “Is always fair to do what technology makes doable?” It is a tormenting question that has accompanied humanity, since from the beginning. Suffice to think about the Greek myth of Prometheus, who defied Zeus by stealing fire and giving it to the humans.

There are technologies involved in productive processes, which affect negatively our environment and seem to induce global warming. Is it fair to quit these processes although they give work to many people? Can we find valid alternatives without creating unemployment?

There are other technologies that can manipulate, reshape, and re-engineer life; they are in perpetual refinement (Metzl 2019; Kozubek 2016; Doudna and Sternberg 2018; Parrington 2016). The rate of technological development is swift, and the governance structures struggle to keep up. Some technologies intervene at the beginning of a new life, and inevitable bioethical discussions arise. These discussions are dominated by questions such as “Is it fair to manipulate embryonic stem cells?”; “Are the techniques of in-vitro fertilization fair?”; “Is abortion acceptable?”; “Is it sure to create genetically modified organisms although we cannot predict all the consequences of their spreading?”. Some technologies can significantly enhance human intellect and physiology. “Should such enhancement technologies be used although they can change the material essence of what has been a human being?” (Rana and Samples 2019). Finally, other bioethical issues concern about suffering and the end of life. The latter problems raise questions such as “Is euthanasia fair?”; “What about therapeutic obstinacy?”; “Is organ donation fair?”; “Is it fair to perform experiments with animals?”.

It is compelling to find universally shared solutions to all these bioethical concerns, but it is tough to achieve them. The difficulties originate from the absence of universal ethical principles and the limits of predictability we have about Complex Systems as humans, ecosystems, and the climate are. All the bioethical issues mentioned here constitute what we might define as Bioethical Complexity (BEC).

## How to tackle the twenty-first century challenges?

**Fig. 3:**
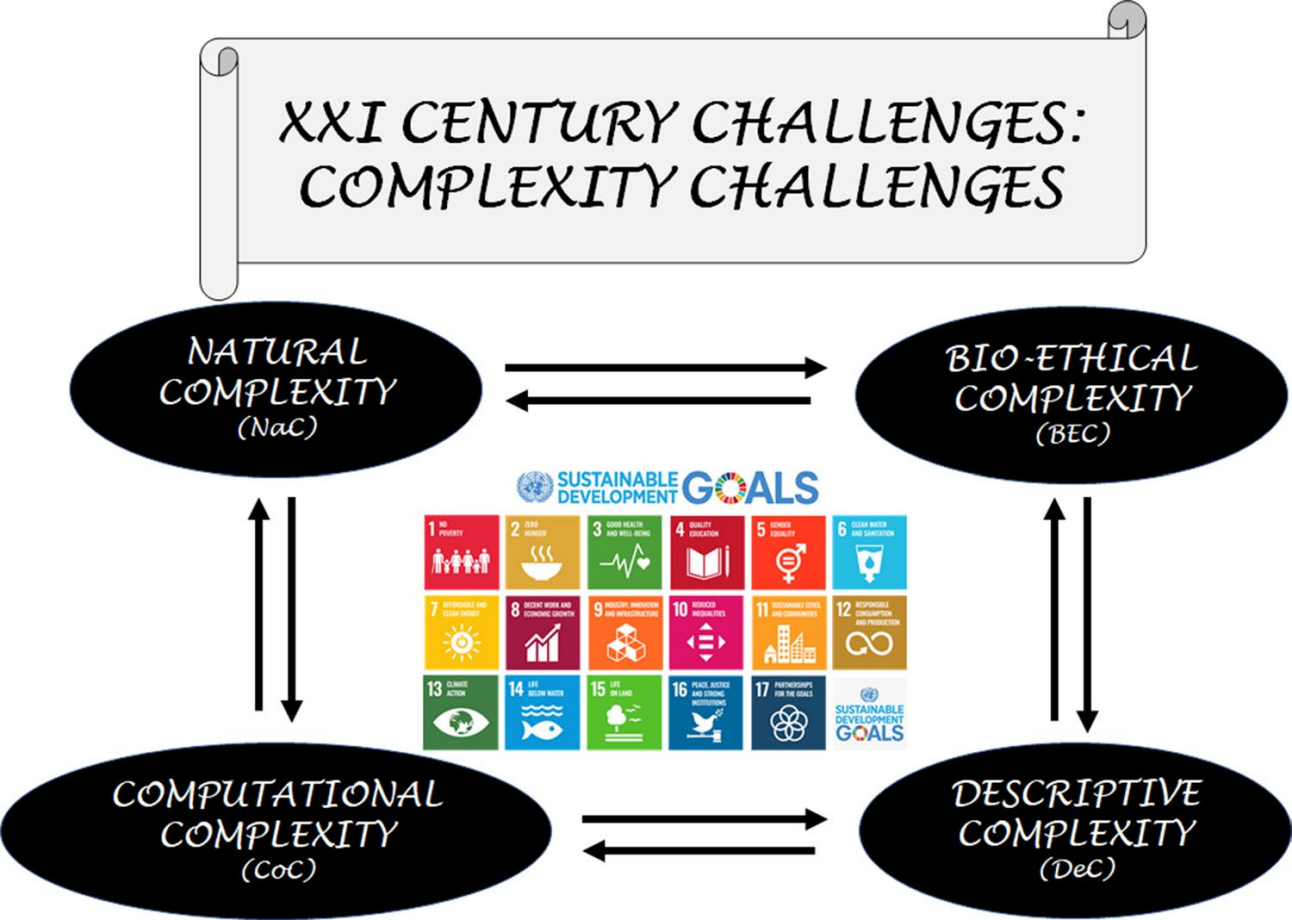
Scheme showing that the challenges of this century, included in the 17 goals of the 2030 Agenda, are Complexity Challenges because they regard Natural Complexity (NaC), Bioethical Complexity (BEC), Computational Complexity (CoC), and Descriptive Complexity (DeC)

Based on what has been stated in this work, it is reasonable to name the challenges included in the 2030 Agenda as Complexity Challenges. They regard Natural Complexity (NaC) and Bioethical Complexity (BEC), which are connected to Computational Complexity (CoC) and Descriptive Complexity (DeC) (see Fig. 3).

Which are promising strategies to tackle the Complexity Challenges? First, we need an update on the methodological approaches to deepen and develop further the Complexity Science.

### New methodological approaches

**Fig. 4:**
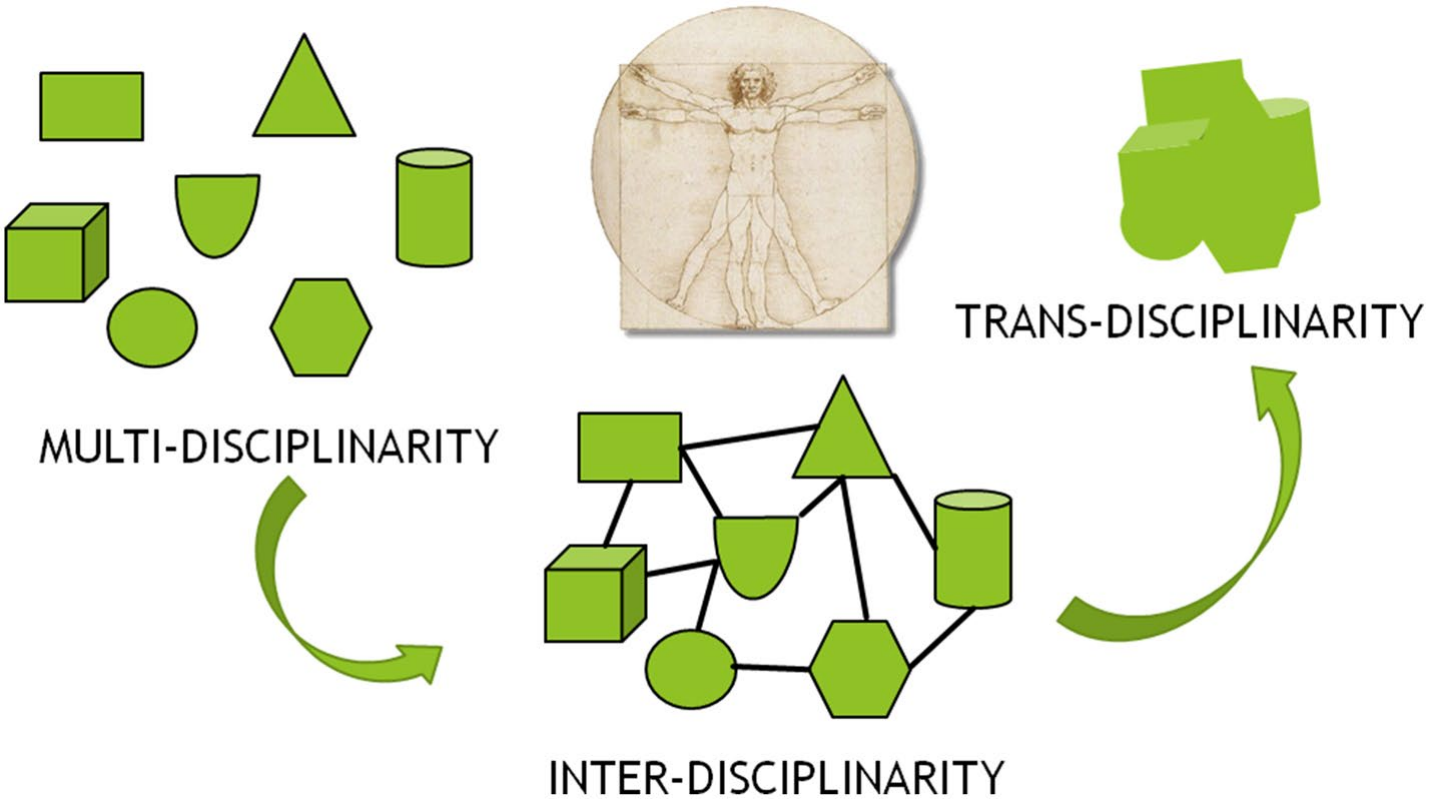
The description of any Complex System, such as a human being represented by the Vitruvian Man, requires to move from a multidisciplinary to an interdisciplinary approach based on Complexity Science. The final goal is to achieve a uniform and comprehensive transdisciplinary image of any Complex System

Complexity Science grounds on multi-disciplinarity; it requires interdisciplinarity, and it targets trans-disciplinarity. Figure 4 depicts the difference. When a Complex System, such as a human being represented by the Vitruvian Man, is described by many distinct disciplines, illustrated as different geometrical figures, then a multidisciplinary, fragmented, and polyhedric picture is generated. When the distinct fields interact and pinpoint the methods and models they share, a disciplinary network is formed, and a more organic description of the Complex System emerges through interdisciplinarity. The final goal is trans-disciplinarity, which will be achieved when the Complexity Science will formulate a uniform description of Complex Systems, breaking down the traditional disciplinary boundaries. This article wants to be a contribution in this direction.

Meanwhile, interdisciplinary efforts are needed to tackle the Complexity Challenges (Morin 2001). The comprehension of Natural Complexity requires dealing with Computational and Descriptive Complexity, as well. All the scientific disciplines must cooperate to succeed: mathematics, computer science, all the natural sciences, along with economic and social sciences. When we face Bioethical Complexity, the involvement of not only scientists but also jurists, philosophers, theologians, and artists is necessary. All forms of art might be beneficial. Artists, guided by their intimate feelings and intuitions, may spark new ideas in human minds and promote the formulation of unconventional ways of reading reality. The basis for fruitful interdisciplinary cooperation must be prepared at school and university. The teaching of separate disciplines, without any intercommunication, produces a fragmentation that forbids an overarching vision of the reality (Morin 2001). Education must be reorganized (Dominici 2018). An initial and easy-to-implement strategy could be that of proposing interdisciplinary courses on Complex Systems in the different faculties (Gentili 2019). The European Commission has recently funded a Strategic Partnership European project Erasmus + titled “Enhancing higher education on COmplex SYstems THINKING for sustainable development”, whose acronym is “COSY THINKING” (Project N°: 2020-1-SE01-KA203-077872). Its final objective is to design an interdisciplinary curriculum focused on the sustainable development required by the 2030 Agenda, and based on Complexity Science.

The study of Complex Systems shows that reductionism, which is a cornerstone of the scientific method, is not enough. A systemic approach is also required. Furthermore, the experimental investigation of Complex Systems reveals that we cannot rely upon the reproducibility of the experiments. Most of the experiments involving Complex Systems are historical events. In this regard, the image that the philosopher Popper (1979) has put forward in his essay “Of clouds and clocks” is particularly appropriate. In the past, science had been occupied with clocks, i.e., simple and deterministic systems having reproducible behavior. On the other hand, nowadays, science has to deal with clouds, i.e., Complex Systems, having unique and hardly replicable behaviors.

The investigation of Complex Systems demands the collection, storage, and elaboration of massive datasets, i.e., the so-called Big Data (Marx 2013). Therefore, it is compelling to contrive smart methods and tools to face the enormous volume and the fast stream of data, their variety (they might have many types of formats), variability, and their relationships. Computer simulations are alternative ways of performing experiments on Complex Systems. It is urgent to accelerate the rate of our computing machines and extend their memory space. New algorithms are inevitably needed to face the Complexity Challenges. There are two promising strategies to succeed (Gentili 2018a, b). One is by improving the electronic computers, and the other is the interdisciplinary research line of Natural Computing.

### Improving electronic computers

The first electronic computers appeared in the 1950s, and their architecture was devised by John Von Neumann (Burks et al. 1963). Although their performances have been improving vertiginously, the architecture has not changed so far. The computer’s principal elements are an active central processing unit (CPU) and a passive memory. The memory stores both the data and the instructions to manipulate the data. Data and instructions are encoded as binary digits through electrical signals, and transistors are the basic switching elements for processing Boolean logic.

Since 1965, the pace of the electronic computers’ improvement has been described by Moore’s law stating that the number of transistors per chip doubles every 2 years (Moore 1995). By miniaturizing the transistors, the voltage needed to power them scales downward, too. The number of transistors per chip increases, and the number of computational steps that can be performed at the same cost grows. Sooner or later, Moore’s law will stop to hold because transistors will be made of a few atoms. Chips’ manufacturers are investing billions of dollars in ideating new computing technologies beyond Moore’s law (Waldrop 2016). One strategy consists in substituting silicon, which is the primary constituent of current transistors, with other materials, such as graphene (Schwierz 2010) and carbon nanotubes (Cavin et al. 2012). Another strategy consists in revolutionizing the hierarchical structure of the memory by introducing memristors. A memristor is an electronic component whose resistance is not constant but depends on how much electric charge has flowed in what direction through it in the past (Yang et al. 2013). Memristors’ cells can be exploited to devise a RAM that is not volatile. A further solution to reduce data-movement demands and data access bottlenecks between the CPU and memory is to extend the electronic devices into a third, vertical dimension by stacking together microprocessors and memory chips (Sabry Aly et al. 2015).

The relentless improvement of electronic computers and cloud computing (Waldrop 2016), which consists of using a worldwide network of remote servers hosted on the Internet to store, manage, and process data, bring benefits to the investigation of Complex Systems. However, there is another promising research line for winning the Complexity Challenges, and it is Natural Computing.

### Natural Computing

Researchers working in the field of Natural Computing (Rozenberg et al. 2012; Gentili 2018a, b; Gentili 2018a, b) draw inspirations from nature to propose:

- New algorithms;
- New materials and architecture to compute;
- New methodologies and models to interpret Natural Complexity.

The rationale is that any distinguishable physicochemical state of matter and energy can be used to encode information; every natural transformation is a kind of computation. Within Natural Computing, two research programs are prominent.

**Fig. 5:**
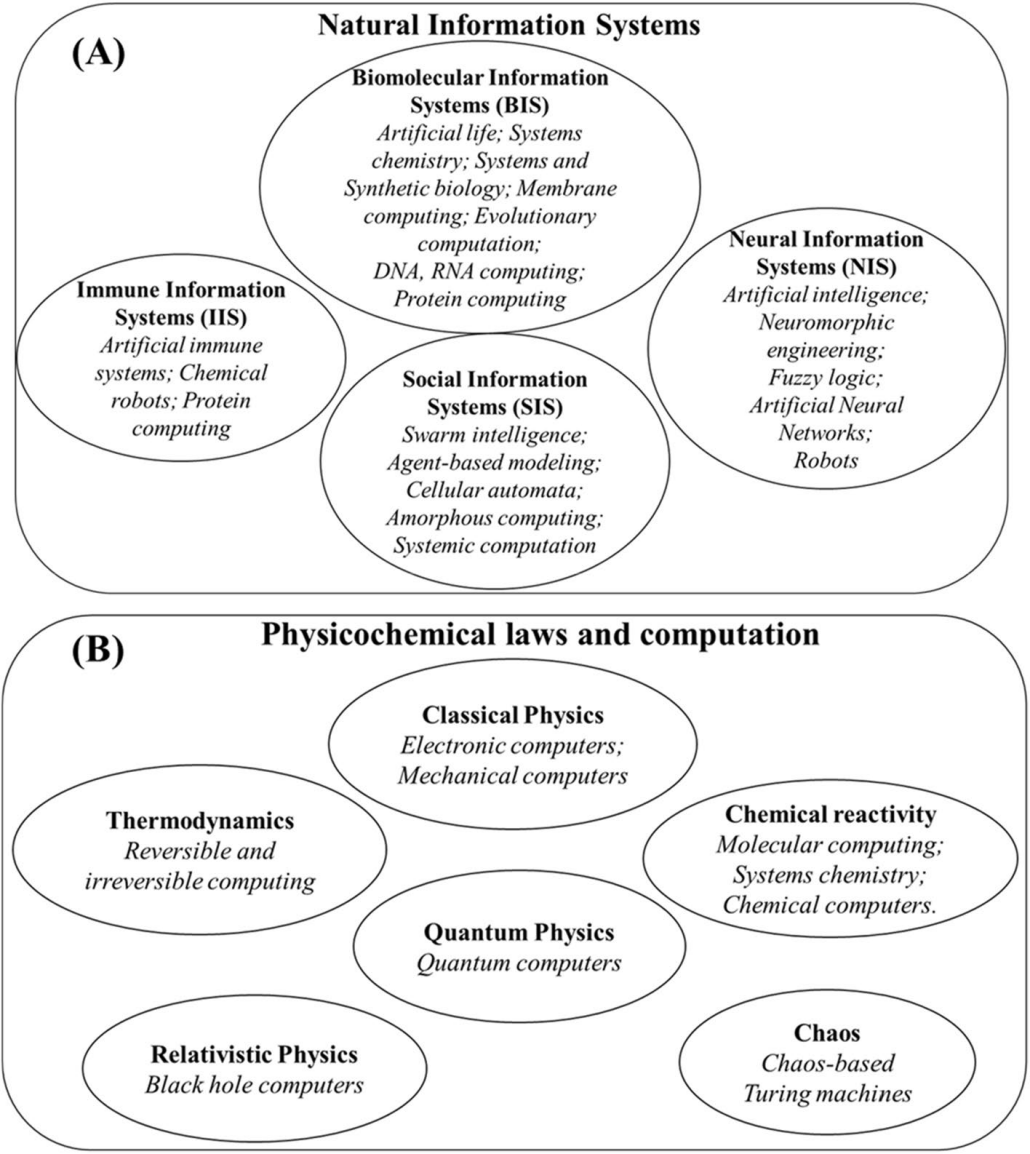
List of the Natural Information Systems and the research lines that derive from their imitation in (**a**); list of the physicochemical laws that can be exploited to make computations in (**b**)

In the first program, scientists mimic the features and the performances of the natural information systems, i.e., all those living systems that exploit matter and energy to encode, collect, store, process, and send information. The natural information systems to imitate are the living cells (also said Biomolecular Information Systems or BIS), the nervous systems (also named as Neural Information Systems or NIS), the immune systems (Immune Information Systems or IIS), and the societies (Social Information Systems or SIS). The mimicry of the natural information systems has promoted the development of different research lines, as shown in Fig. 5a.

In the second program, researchers exploit the physicochemical law to make computations (see Fig. 5b). Every physicochemical law describes a causal event, and any causal event can be conceived as a computation. The causes are the inputs, the effects are the outputs, and the law governing the transformation is the algorithm of the computation.

In agreement with the rationale of Natural Computing, the cognitive scientists Gallistel and King (2010) and the neuroscientist Marr (2010) have proposed a methodology to describe and understand Complex Systems. Such methodology requires an analysis of any Complex System at three levels. The first analysis is at the “computational level”. It consists in describing its transformations as if they were computations after determining the inputs and the outputs. The second analysis is at the “algorithmic level”. It consists in formulating algorithms that might carry out the computations pointed out in the previous level of analysis. The third and conclusive analysis is at the “implementation level” and consists of looking for mechanisms to implement the algorithms. If the analyses at the three levels have been conducted properly, the mechanism found in the final analysis will be a plausible replication of the Complex System’s behavior. Hopefully, this methodology will allow us to better understand Complex Systems and it will give us new tools to reach the goals of the 2030 Agenda. It will likely promote the formulation of a brand new transdisciplinary theory concerning Natural Complexity (Gentili 2018a, b). Based on what is looming, this transdisciplinary theory will have information as its key-variable. Information will be outlined not only from the quantitative but also the qualitative, i.e., semantic point of view. Such expected theory will presumably give some clues about that mysterious event, which was the appearance of life on Earth roughly 3.5 billion years ago. The appearance of life on Earth was like a “phase transition” (Walker et al. 2017) from inanimate chemical systems, driven just by force fields, to the living chemical systems, able to exploit the matter and energy to encode, process, send, and store information. A clarification of this enigmatic event will have repercussions in our attempts to face Bioethical Complexity and develop new technologies, such as Chemical Artificial Intelligence (Gentili et al. 2016, 2017; Bartolomei et al. 2020).

## Conclusions

Do we need to investigate Complex Systems? The answer is, undoubtedly, yes. Paraphrasing what the famous German mathematician David Hilbert (1862–1943 AD) was used to say: As long as a branch of science tries to face an abundance of problems, “so long it is alive; a lack of problems foreshadows extinction or the cessation of independent development.” The Complexity Science is particularly alive because it is indispensable when facing the challenges included in the 2030 Agenda compiled by the United Nations. This article is a contribution to the development of Complexity Science because it proposes a list of the features shared by all the Complex Systems involved in the 2030 Agenda and a preliminary transdisciplinary definition of Natural Complexity. Reaching the goals of the 2030 Agenda is a tough task due to the limited predictability of the Complex Systems. The reasons why the behavior of Complex Systems is hard to be predicted in the long term are explained in this article. The awareness of limited predictability of Complex Systems makes many cutting-edge technologies highly disputable from an ethical point of view: those technologies affecting the climate and those manipulating life in particular. Humanity cannot ignore dealing with Bioethical Complexity. However, to win all the challenges of this century, included those listed in the 2030 Agenda, it is necessary to update the methodologies, as explained in this article. In particular, the high and massive barrier separating the scientific and humanistic knowledge should be demolished to reach a genuinely transdisciplinary perspective of complexity. Finally, Natural Complexity might be partly untangled if we successfully face Computational and Descriptive Complexity. In this regard, it is urgent to improve our computing machines’ performances and contrive new ones, pursuing the research line of Natural Computing. These efforts will bring to the formulation of a new theory on Complex Systems (Caldarelli et al. 2018) with relevant benefits for the sustainable development of our world.

## Footnotes

1 The brute force solution of the TSP requires an exhaustive search that consists in measuring the length of all the permutations of edges and, then, seeing which is the cheapest.

2 Also said Kolmogorov Complexity.
